# Neighbourhood physical activity environments and adiposity in children and mothers: a three-year longitudinal study

**DOI:** 10.1186/1479-5868-7-18

**Published:** 2010-02-19

**Authors:** Anna Timperio, Robert W Jeffery, David Crawford, Rebecca Roberts, Billie Giles-Corti, Kylie Ball

**Affiliations:** 1 Centre for Physical Activity & Nutrition Research, School of Exercise & Nutrition Sciences, Deakin University, 221 Burwood Hwy, Burwood VIC 3125, Australia; 2 Obesity Prevention Center, Division of Epidemiology and Community Health, University of Minnesota, 1300 S 2nd Street, Suite 300, Minneapolis, MN 55454, USA; 3 School of Population Health, The University of Western Australia, 35 Stirling Hwy, Crawley WA 6009, Australia

## Abstract

**Background:**

Although neighbourhood environments are often blamed for contributing to rising levels of obesity, current evidence is based predominantly on cross-sectional samples. This study examined associations between objectively-measured environmental characteristics of neighbourhoods and adiposity cross-sectionally and longitudinally over three years in children and their female carers.

**Methods:**

Longitudinal study of 140 5-6 year-old and 269 10-12 year-old children and their female carers (n = 369). At baseline (2001) and follow-up (2004), height and weight were measured among children and self-reported among female carers, and were used to compute BMI z-scores and BMI, respectively. A Geographic Information System determined access to destinations (public open spaces, sports options, walking/cycling tracks), road connectivity (density of cul-de-sacs and intersections, proportion of 4-way intersections, length of 'access' paths (overpasses, access lanes, throughways between buildings)) and traffic exposure (length of 'busy' and 'local' roads) within 800 m and 2 km of home. Univariate and multivariable linear regression analyses examined associations between environmental characteristics and BMI/BMI z-scores at baseline and change in BMI/BMI z-scores over the three years.

**Results:**

Cross-sectionally, BMI z-score was inversely associated with length (km) of access paths within 800 m (b = -0.50) and 2 km (b = -0.16) among younger and number of sport/recreation public open spaces (b = -0.14) and length (km) of 'access' paths (b = -0.94) within 800 m and length of local roads within 2 km (b = -0.01) among older children. Among female carers, BMI was associated with length (km) of walking/cycling tracks (b = 0.17) and busy roads (b = -0.34) within 800 m. Longitudinally, the proportion of intersections that were 4-way (b = -0.01) within 800 m of home was negatively associated with change in BMI z-score among younger children, while length (km) of access paths (b = 0.18) within 800 m was significant among older children. Among female carers, options for aerobics/fitness and swimming within 2 km were associated with change in BMI (B = -0.42).

**Conclusion:**

A small number of neighbourhood environment features were associated with adiposity outcomes. These differed by age group and neighbourhood scale (800 m and 2 km) and were inconsistent between cross-sectional and longitudinal findings. However, the results suggest that improvements to road connectivity and slowing traffic and provision of facilities for leisure activities popular among women may support obesity prevention efforts.

## Background

Obesity is an increasingly prevalent health condition in Australia. Almost one in four Australian children and adolescents [1] and three in five Australian adults [2] are overweight or obese. The rate of recent changes in obesity in Australia and other parts of the world has led many to conclude that changes to the environment, rather than genetic or personal factors, are most likely to be responsible [3-5]. The impact of the environment on obesity is likely to be mediated through key behaviours that contribute to energy imbalance, such as physical activity, sedentariness and eating behaviours [6]. Thus, environmental factors likely to have the most leverage are those that reduce the need and/or opportunity to expend energy through physical activity, those that encourage sedentary behaviours and those that increase opportunities and incentives for energy intake through eating. Environmental factors that have been shown to negatively influence physical activity behaviours include limited access to recreational facilities, such as parks, sports venues, sidewalks, bicycle/walking trails [7,8], and environmental characteristics that promote automobile use and discourage walking for transportation, such as road systems that lack interconnectivity, low neighbourhood walkability, lack of access to public transportation and unsafe streets due to traffic and/or crime [7,9,10].

Despite the attention given to the role of such environmental factors on obesity in the scientific and lay press, empirical research on the topic is limited predominantly to cross-sectional studies and the results to date are mixed. A review by Papas and colleagues [6] in 2007 identified 12 studies that examined objectively-assessed features of the built environment related to physical activity and risk of obesity in *adult *populations. These studies were all cross-sectional or ecological and there was much disparity between studies in the types of features examined, measurement methods and the scale or size of the neighbourhood studied (i.e., how neighbourhoods were defined). However, there were associations found between many aspects of the built environment and obesity risk, such as distance and access to facilities, sidewalk availability, neighbourhood walkability, sprawl, mixed land use, greenery, graffiti and population density. More recent studies have also shown associations between obesity and lack of trails, high traffic, greater number of neighbourhood barriers, street connectivity and amenities [11,12].

Among youth, a recent review of 15 studies (including only one longitudinal study) examining features of the physical environment and adiposity [13] reported an inconsistent pattern of findings. Among children, results differed by sex, age and area of residence. Positive associations with obesity outcomes were found for number of neighbourhood hazards (low socioeconomic areas) and parent perceptions of heavy traffic (older children), while there were negative associations for the amount of vegetation (highly populated areas only), intersection density (girls), child (but not parent) reported access to physical activity facilities and bike/walking trails and objectively-assessed neighbourhood walkability (girls). Among adolescents, obesity was associated with access to facilities, neighbourhood type and urban sprawl [13].

To date, the existing literature has failed to identify strong and consistent physical activity-related environmental correlates of obesity cross-sectionally. In part, this may be because there has been little consistency across studies in the way in which both neighbourhoods and neighbourhood features are defined and most studies focus on only few features. There is also a dearth of longitudinal studies [6,13] and none to date that have examined both children and adults simultaneously [6]. The present investigation sought to add to the current evidence base by examining cross-sectional and longitudinal associations between objectively-measured physical activity-related neighbourhood characteristics and adiposity among children and their female carers.

## Methods

The Children Living in Active Neighbourhoods (CLAN) Study is a longitudinal study that examined family and neighbourhood influences on children's physical activity and sedentary behaviour. Two age cohorts, 5-6 and 10-12 year-old children, and their parents were recruited in 2001 (baseline) and followed up in 2004 (follow-up). Parents of children in each cohort completed a self-administered questionnaire. The Deakin University Human Ethics Committee and the Department of Education and Training Victoria approved baseline and follow-up data collection protocols. The Catholic Education Office also approved the data collection protocols for follow-up.

### Sample

Families of children in both age cohorts were recruited from 19 state elementary schools in Melbourne, Australia in 2001. Details of baseline recruitment have been described previously [14-16]. Briefly, 698 families agreed to be recontacted for further research and were subsequently invited to participate in the three-year follow-up study (2004). At both baseline and follow-up, active consent was required from parents for their own and for their child's participation. In total, 188 5-6 year-old and 402 10-12 year-olds participated in the follow-up; however the data presented in this paper are based on children and female carers with height and weight data (self-reported for female carers) at each time point and who did not change residential address between 2001 and 2004. Female carers whose partner completed the questionnaire were excluded as proxy-reports of weight may not be accurate [17]. The average length of follow-up for these children was 2.9 ± 0.4 years.

### Measures

#### Adiposity

Children's height and weight were measured without shoes and in light clothing at the child's school at baseline and at the child's school or home at follow-up using a portable stadiometer and digital scale. At follow-up, parents measured the height and weight of 15 children (3.7% of sample) whom we were unable to visit. Body mass index (BMI, kg/m^2^) was computed and converted to age and sex-specific BMI z-scores [18]. Parents reported their own height (without shoes) and weight (without clothes or shoes). Self-reported height and weight values have been shown to be highly correlated with measured values in adult populations [19]. BMI (kg/m^2^) was computed for female carers at each time point. Changes in children's BMI z-scores and female carers' BMI were computed by subtracting follow-up from baseline values.

#### Socio-demographic information

Parents were asked to provide the following socio-demographic information for themselves and their partner (where applicable): age; highest level of education; usually speak English in the home; marital status; and employment status.

#### Neighbourhood environment

A Geographic Information System (GIS) was used to generate objective measures of the neighbourhood physical activity environment (ESRI ArcView 3.3 and related extensions). The GIS dataset included cadastral and road/road infrastructure data supplied by the State of Victoria (VicMap Property, VicMap Address and VicMap Transport). The Open Space 2002 spatial dataset (owned by the Australian Research Centre for Urban Ecology) was also used and walking/cycling paths and access paths were identified using VicMap Transport (Jan 2004) and Melway Map Images (Edition 31, October 2003, Ausway Publishing). Individual participant addresses were geocoded and physical activity destinations, road connectivity and traffic exposure within an 800 m and a 2 km Euclidian buffer of each address point were examined. There is currently no consensus as to how to define a neighbourhood for either children or adults [20,21]. At baseline, parents reported their child's 'walking distance' as 800 m [22]. While this may approximate an appropriate neighbourhood scale for children, walking distances for adults (and for children entering adolescence) are likely to be greater. Moreover, the scale of neighbourhoods may be further extended through vehicle use [23], including parents driving their children to physical activity opportunities [24,25]. Thus two definitions (800 m and 2 km) were used.

##### Destinations

Within each buffer, the density of freely accessible public open spaces (no fees or restricted opening hours) and public open spaces classified as 'sport/recreation', the number of different popular sports available to children (range 0-9), the existence of gyms/leisure centres, swimming pools or both (coded 0-2), and the total length (km) of walking and cycling tracks (excluding overpasses and access lanes/throughways between buildings) were computed. Sports popular among children were defined as those with greater than 5% participation (males and females combined) among 5-14 year-olds in 2003 (basketball, BMX riding, cricket, football, gym, netball, swimming, skating, soccer and tennis) [26]. As aerobics/fitness and swimming are the most popular sports/physical activities among women (excluding walking) [27], the option within the neighbourhood to visit a gym/leisure centre and a swimming pool were examined separately. Locations where each activity/sport could be performed were sourced from community directories, local government, electronic telephone directories and other websites. In addition, distance to school was calculated using the shortest possible route along the road network [22].

##### Road connectivity

Measures within each buffer included number of cul-de-sacs, intersection density, the proportion of intersections that were at least 4-way and the total length (km) of 'access' paths (defined as overpasses, access lanes and throughways between buildings).

##### Traffic exposure

Within each buffer, the total length of roads classified as freeways, highways or arterial roads ('busy' roads with higher speed limits [22]) and of those classified as 'local' roads [28] were computed.

### Statistical analyses

Statistical analyses were conducted using STATA 8.0 (Stata Corporation, College Station, 2003). In each age group, independent t-tests were used to compare mean differences between boys and girls in baseline, follow-up and change in BMI z-score, and in neighbourhood environment exposures. Paired t-tests were used to examine changes in BMI and in BMI z-score between baseline and follow-up for female carers and for boys and girls in each age group, respectively. Each neighbourhood environment variable was entered into separate linear regression models predicting baseline BMI z-score and change in BMI z-score among children and baseline BMI and change in BMI among female carers. Variables significantly related to the outcomes were added simultaneously into multivariable linear regression models after potential collinearity was checked (correlations between entered variables were < 0.7 and the Variance Inflation Factors <2). Regression analyses were conducted separately for 5-6 and 10-12 year-old children (controlling for sex), longitudinal models also adjusted for baseline adiposity and all models were adjusted for clustering by baseline school using robust standard errors. The number of different popular sports available to children was not examined among female carers and the existence of gyms/leisure centres/swimming pools was not examined among children.

## Results

### Sample profile

Data were available for 140 children initially aged 5-6 years (53% boys) and 269 children initially aged 10-12 years 45% boys). Most children had female carers with a university or tertiary qualification (44%). The families of most children usually spoke English at home (97%), and most had parents who were married or living as married (88%). Almost two-thirds (64%) of children had a female carer who was employed full or part-time. Data were available for 369 female carers. Their average age at baseline was 40.1 (SD = 5.3) years. The adiposity of children included in analyses did not differ from other children in the original baseline sample, however a higher proportion included in these analyses (p < 0.05) had parents who were married/living as married, a female carer with a university or tertiary qualification and a family that usually spoke English at home. These differences were also apparent among female carers included in analyses compared to other women in the baseline sample, but there were no differences in BMI or weight status among these women.

### Weight change

BMI z-score remained relatively stable over the three year period for boys. It decreased among younger girls and increased among older girls (Table 1). The mean BMI of female carers increased significantly over the three year period (p < 0.001).

**Table 1:** Mean (SD) BMI z-score and BMI among children and female carers

	5-6 years^a^		10-12 years^a^		Female carer^b^ *(n = 369)*
			
	Boys *(n = 74)*	Girls *(n = 66)*	Boys *(n = 120)*	Girls *(n = 149)*	
BMI z-score/BMI					
Baseline	0.46 (0.94)	0.69 (0.79)	0.44 (0.97)	0.25 (0.91)^e^	24.38 (4.38)
Follow-up	0.47 (0.95)	0.51 (0.85)	0.50 (0.97)	0.45 (0.85)	25.01 (4.69)
p-value^c^	0.836	0.001	0.273	<0.001	<0.001
					
Change in BMI z-score/BMI	0.01 (0.50)	-0.18 (0.41)^d^	0.05 (0.53)	0.20 (0.49)^de^	0.62 (2.61)

^a^BMI z-score (children)

^b^BMI (female carers)

^c^Paired t-test between baseline and follow-up

^d^Independent t-test between boys and girls: p < 0.05

^e^Independent t-test between age groups: p < 0.001

BMI, body mass index

### The neighbourhood environment

Table 2 shows the distribution of objective measures of the neighbourhood environment and highlights variation in exposures.

**Table 2:** Distribution of neighbourhood environment variables among children and female carers

	800 m			2 km		
		
	Children (mean, sd)	Female carers (mean, sd)	Range	Children (mean, sd)	Female carers (mean, sd)	Range
(*n*)	*(409)*	*(369)*		*(409)*	*(369)*	
						
**Destinations**						
Density freely available POS	7.7 (3.7)	7.7 (3.7)	0 -- 20	40.8 (14.4)	40.6 (14.2)	0 -- 79
Density sport/recreation POS	1.0 (0.9)	1.0 (0.9)	0 - 4	5.8 (2.9)	5.8 (2.9)	0 -- 15
Number sport options -- children	2.3 (1.9)	-	0 -- 8	5.7 (1.8)	-	0 -- 9
Number gym/swimming options	-	0.4 (0.7)	0 - 2	-	1.4 (0.8)	0 - 2
Linear km of walk/cycle tracks	3.3 (3.3)	3.3 (3.3)	0 -- 21.6	15.6 (9.1)	15.4 (9.0)	0 -- 60
Distance to school (km)	2.1 (3.2)	2.2 (3.4)	0.04 -- 36.5	-	-	-
						
**Road connectivity**						
Number of intersections	89.2 (38.7)	89.9 (39.5)	1 - 279	480.3 (189.2)	483.0 (194.6)	24 -- 1225
Proportion of 4-way intersections	10.2 (7.7)	10.4 (7.9)	0 - 44.4	10.7 (6.6)	10.8 (6.7)	0 - 25.3
No. of cul-de-sacs	29.6 (17.4)	29.3 (17.6)	1 - 97	156.3 (73.9)	153.1 (72.4)	12 -- 402
Linear km of 'access' paths	0.2 (0.3)	0.2 (0.3)	0 - 1.4	1.2 (0.9)	1.2 (0.9)	0 - 4.9
						
**Traffic exposure**						
Total length of 'busy' roads (km)	1.9 (1.7)	1.9 (1.7)	0 - 7.9	12.0 (6.9)	12.0 (6.7)	0 - 30.5
Total length of local roads (km)	16.0 (4.8)	16.1 (4.8)	1.0 -- 36.6	87.3 (25.8)	87.8 (26.4)	8.4 - 144.7

### Cross-sectional associations with BMI

Only length of access paths (km) within 800 m of home was inversely associated with BMI z-scores among younger children (B = -0.50, 95%CI = -0.84,-0.16, p = 0.006). Among older children, length of access tracks (B = -0.54, 95%CI = -0.94,-0.13, p = 0.041) and number of sport/recreation public open spaces (B = -0.14, 95%CI = -0.26,-0.10, p = 0.033) were inversely related to BMI z-scores in both univariate and multivariable analyses (values for multivariable analyses only reported here). Consistent with univariate models, among female carers length of walking/cycling tracks within 800 m was positively (b = 0.17, 95%CI = 0.09, 0.26, p < 0.001) and length of busy roads within 800 m negatively (b = -0.34, 95%CI = -0.60, -0.08, p = 0.012) associated with BMI in multivariable analyses.

Using a 2 km buffer around participant homes, access paths was again significantly associated with BMI z-score among younger (B = -0.16, 95%CI = -0.30,-0.02, p = 0.027) but not older children. Length (km) of local roads within 2 km of home was negatively associated with BMI z-scores among older children (B = -0.01, 95%CI = -0.01,-0.00, p = 0.048). None of the neighbourhood features within 2 km were associated with BMI among female carers.

### Longitudinal associations with BMI

Intersection density (B = -0.002, 95%CI = -0.003,-0.0003, p = 0.02) and the proportion of 4-way intersections (B = -0.01, 95%CI = -0.02,-0.003, p = 0.012) within 800 m of home were inversely associated with change in BMI z-score among younger children, but only number of 4-way intersections remained significant in the multivariable model (B = -0.01, 95%CI = -0.01,-0.0002, p = 0.044). Only length of access paths (B = 0.18, 95%CI = 0.01,0.35, p = 0.04) within 800 m was associated with greater relative increases in BMI z-score among older children. There were no associations between BMI z-scores and neighbourhood features within 2 km among children. However, among female carers the number of options for aerobics/fitness and swimming within 2 km was associated with greater relative decreases in BMI (B = -0.42, 95%CI = -0.83, -.0005, p = 0.05).

## Discussion

This study is one of the first to examine associations between the neighbourhood physical activity environment and adiposity longitudinally, and to examine both children and adults simultaneously. Overall, few objectively assessed characteristics of the neighbourhood environment included in this study were associated with adiposity or changes in adiposity over three years among children in early and later primary school or among their female carers. Features for which there were associations generally differed by age group and there was little consistency between cross-sectional and longitudinal findings, and between the two neighbourhood scales (800 m and 2 km) adopted in this study. This suggests that different factors may be important during different life stages [20], and highlights the importance of careful consideration of the scale used for studies of different age groups and different behaviours. Nevertheless, in general, most associations were found for features within 800 m of home, rather than features within the wider definition of a neighbourhood (2 km), regardless of age.

Access to destinations for physical activity was found to be associated with adiposity among older children and female carers, but not younger children. The density of public open spaces designated specifically as sport/recreation was negatively associated with BMI z-scores among older children, suggesting that the provision of spaces specifically for physical activity may be important in efforts to prevent obesity in children. This finding is consistent with previous research linking access to recreational facilities with physical activity among children [7]. Notably, the number of different sports that are popular among children that were available within the local neighbourhood was not associated with adiposity in any analyses. This measure, however, does not capture less organised forms of physical activity that may be important among children. These findings should be considered with caution since the cross-sectional finding regarding sport/recreation spaces was not replicated in the longitudinal analyses. This may reflect changes in leisure interests as children age, whereby these kinds of sports and recreation facilities may be less appropriate, relevant or desirable to mid-adolescents than to 10-12 year-olds. Consistent with the literature among adults [29], access to gyms/leisure centres and swimming pools within 2 km was associated with relatively smaller increases in BMI in longitudinal analyses among female carers. Provision of facilities for leisure activities popular among women, combined with consideration about the cost of using those facilities particularly in disadvantaged areas [30], may therefore be an important strategy for obesity prevention. However, cross-sectionally, length of walking/cycling tracks within 800 m of home was associated with higher BMI among women in this study - an unexpected finding that requires further exploration.

Road connectivity is thought to be an important component of the walkability of neighbourhoods because neighbourhoods that are better connected reduce travel times by foot or cycle and offer a greater diversity of routes to local destinations without adding substantially to travel times [9]. In this study, intersection density and proportion of intersections that were at least 4-way, both indicators of connectivity [31], were longitudinally associated with relatively greater decreases in BMI z-scores over the three years among younger children, but not among older children. Similar findings have been found among children of a similar age in Canada [32], while Frank and colleagues [33] found intersection density to be a correlate of walking behaviour among adolescents but not children. Few studies have examined associations between similar measures of connectivity and physical activity behaviour among young children [7]. More research is needed to better understand relationships between connectivity and adiposity in this age group and mediators of this relationship. Although connectivity is a well-known correlate of walking behaviour among adults [9], no associations were found with adiposity outcomes among female carers in this sample. Length of access paths is also a measure of connectivity, since these paths facilitate pedestrian travel by going under or over major roads, and making it quicker and less dangerous to reach destinations [34]. In this study, access paths were negatively associated with BMI z-scores among both groups of children, suggesting that living in areas with many shortcuts or cut-throughs is important among children, possibly making it safer for children to walk locally. However, access paths were not associated with change in BMI z-score longitudinally in the younger children and became associated with greater relative increases in BMI z-scores among older children. This inconsistent finding needs further study.

Although the total amount of 'busy' roads within neighbourhoods was unrelated to adiposity outcomes in children, cross-sectionally greater length of local roads were associated with lower BMI z-scores among the older children. This finding is consistent with earlier cross-sectional findings in the same sample of children where the older children, but not the younger children, were more likely to be overweight or obese if their parents perceived there to be heavy traffic in their local streets and more likely to be obese if their parents agreed that road safety was a concern [14]. Given that older children have greater independence, are more likely to walk or cycle in their neighbourhoods [16,33] and are therefore more likely to be exposed to traffic, it is not surprising that associations were found for older children and not younger children who may be more likely to be accompanied by an adult or driven. These findings suggest that efforts to slow traffic in residential areas may be important for obesity prevention in children. However, again, this cross-sectional finding was not replicated longitudinally. One possible explanation is that as children age they gain greater competence as pedestrians or cyclists and traffic levels may become less relevant. They may also be more likely to use streets for travel rather than as a venue for play where traffic levels would be of greater importance. In contrast to the cross-sectional findings among children, length of busy roads was negatively associated with BMI among female carers. This may be because areas with more busy roads reflect a more connected street network, though there were no associations with other measures of connectivity in this study. Alternatively, the negative association between length of busy roads and BMI among female carers may be confounded by area-level socioeconomic status, whereby areas with busier roads may be more likely to be situated in inner-city areas and may potentially have higher levels of advantage. The relationship between socioeconomic status and obesity risk is well documented [35,36].

The strengths of the present study include the use of a range of objective indices of environmental variables thought to influence physical activity, its prospective design, its use of unique neighbourhoods specific to individual residential addresses rather than administrative definitions [21], and the inclusion of children and adults exposed to the same neighbourhoods. The latter strength, however, also poses challenges in defining 'neighbourhoods' and thus two definitions were used here to accommodate both children and adults. It is possible that the 2 km radius may have been too large, even for adults, and may have attenuated any impact of the local environment on obesity, which may explain the null relationships between many of the attributes of the environment examined here and adiposity using this neighbourhood definition. In adults, for example, it has been suggested that the 'neighbourhood' be restricted to a 15 minute walk of 1.6 km [20]. Given parental concern for children's safety, however, a neighbourhood that might encourage children's physical activity may be considerably smaller. Indeed, more associations were found for features measured within 800 m than within 2 km of home.

The study is limited by the relatively small sample size. Further, this study examined only physical features of the environment; it is possible that other features of the environment for which we do not have archival quantitative measures, such as the quality of destinations, may be stronger determinants of obesity. Moreover, perceptions of the environment may be stronger predictors of behaviour than objective variables [37]. In addition, given that relatively few significant associations were found, it is possible that some associations may have been due to chance given the large number of statistical tests that were conducted for each age group. The results may also be masked by gender differences that were unable to be explored due to the small sample sizes. Although the longitudinal design is a strength, the lack of consistency between cross-sectional and longitudinal findings requires further exploration and may be due to changes in the saliency or importance of specific neighbourhood features as individuals age, mature and gain greater autonomy over what could be considered a substantial period of time among children. Alternatively, it is possible that the neighbourhood environment may have undergone change during the three years to follow-up. These issues should be considered in future prospective studies in order to establish causality. Finally, this study did not consider attributes of the environment that may be related to the other side of the energy balance equation, energy intake. Future studies should simultaneously consider the impact of neighbourhood physical activity and food environments on adiposity.

## Conclusions

This study found few associations between objectively assessed characteristics of the neighbourhood environment and adiposity outcomes, with differences by age group and inconsistencies between cross-sectional and longitudinal findings. However, the results suggest that the environment most proximal to residential areas may be most important in both adults and children. In particular, improvements to road connectivity and slowing traffic may be important strategies to support obesity prevention efforts. Although findings among children were generally in the expected direction, some findings among female carers were not as expected and require further exploration. Further research should also consider examining perceptions of neighbourhood environments, potential changes in neighbourhood environments over time and the role of the neighbourhood food environment in relation to adiposity using a prospective design.

## Competing interests

The authors declare that they have no competing interests.

## Authors' contributions

AT and RWJ conceived of and each drafted sections of the paper and AT performed data analyses. AT, DC, BGC and KB participated in the design of the CLAN study. RR was responsible for generating all spatial data. All authors assisted in the interpretation of the data, provided critical feedback on drafts and read and approved the final manuscript.
